# The relationship between chiropractor required and current level of business knowledge

**DOI:** 10.1186/s12998-017-0134-2

**Published:** 2017-02-06

**Authors:** Michael Anthony Ciolfi, Patsy Anne Kasen

**Affiliations:** 1 University of Bridgeport, 126 Park Ave, Bridgeport, CT 06604 USA; 2 Walden University, 100 S Washington Ave #900, Minneapolis, MN 5541 USA

**Keywords:** Chiropractic, Organizational behavior, Strategic management, Finance, Marketing, Ethics, Accounting, Decision making, Operations

## Abstract

**Background:**

Chiropractors frequently practice within health care systems requiring the business acumen of an entrepreneur. However, some chiropractors do not know the relationship between the level of business knowledge required for practice success and their current level of business knowledge. The purpose of this quantitative study was to examine the relationship between chiropractors’ perceived level of business knowledge required and their perceived level of current business knowledge.

**Methods:**

Two hundred and seventy-four participants completed an online survey (Health Care Training and Education Needs Survey) which included eight key business items. Participants rated the level of perceived business knowledge required (Part I) and their current perceived level of knowledge (Part II) for the same eight items. Data was collected from November 27, 2013 to December 18, 2013. Data were analyzed using Spearman’s ranked correlation to determine the statistically significant relationships for the perceived level of knowledge required and the perceived current level of knowledge for each of the paired eight items from Parts I and II of the survey. Wilcoxon Signed Ranks Tests were performed to determine the statistical difference between the paired items.

**Results:**

The results of Spearman’s correlation testing indicated a statistically significant (*p* < 0.01) positive correlation for the perceived level of knowledge required and perceived current level of knowledge for six variables: (a) organizational behavior, (b) strategic management, (c) marketing, (d) legal and ethical, (e) managerial decisions, and (f) operations. Wilcoxon Signed Ranks testing indicated a significant difference for three paired items: strategic management; marketing and; legal and ethical. The results suggest that relationships exist for the majority of business items (6 of 8) however a statistically difference was demonstrated in only three of the paired business items.

**Conclusion:**

The implications of this study for social change include the potential to improve chiropractors’ business knowledge and skills, enable practice success, enhance health services delivery and positively influence the profession as a viable career.

## Background

The purpose of this study was to examine the relationship between chiropractors’ perceived level of business knowledge required and their perceived level of current business knowledge. Chiropractic has been identified as the largest profession of primary health care providers under the umbrella of complementary and alternative medicine (CAM) [1]. The chiropractic profession offers health care services utilizing natural healing through a core competency of spinal manipulative therapy (SMT) as well as other adjunctive therapies and strategies. The public perceives the profession within a narrow scope of practice while inter-professional consensus favors a nonsurgical spine-specialist practice identity [2]. Chiropractic educational institutions concentrate on SMT and other primary contact patient care with limited business and marketing skills [3]. From a patient perspective, limited knowledge of the chiropractic profession can result in limited utilization rates and practice success. Primary contact health care providers play an essential role to meet the health care demands of society into the 21st century in an environment of health care reform [4].

Chiropractic is the most identifiable CAM primary portal of entry profession in the United States [5]. A primary portal of entry health care provider includes the health care professionals that can examine, diagnose, and provide care directly to a patient, without referral from another health care provider. Factors influencing the practitioner/patient relationship can include educational items such as business, marketing, public health, healthy lifestyles, and collaborating with other health care professionals [3, 6]. Health care providers and chiropractors that successfully market their health care services can provide valuable information and options for patient care in line with the goals of Healthy People 2010/2020 [7]. Healthy People 2010/2020 is a science-based, 10-year national objective program designed to provide target goals to improve the health of Americans [8]. Improved health information allows patients to make informed decisions towards preventative health care aligned with meeting the needs of sustainable and affordable health care objectives [9].

Health care systems provide societies with the ability to maintain the health status of the population, thereby contributing to the overall economic status of the nation. Originally designed to meet the needs of society in the 1960s, the health care systems of North America developed following post World War II periods of growth [10]. In the United States, the inclusion of Medicare and Medicaid occurred in 1965 as part of the Social Security Act [11]. After more than five decades, the original sustainability of these programs is questionable [10]. The governments of North America have provided health coverage for the poor and chronically ill through social programs and taxation. Private health insurance is designed to provide health care coverage to those with adequate financial resources. Despite these measures, the general health of the population of North America has declined (especially for conditions related to lifestyle) with significant increases in obesity and diabetes [12, 13]. An unhealthy society places pressure on employers and governments to provide increased health care services as a greater number of individuals, at a younger age, must utilize governmental and/or private insurance health care systems [14]. Chronic conditions that arise at a young age can increase the need for the individual to access health care services over their lifetime.

To encourage change, a paradigm shift towards wellness, prevention, and greater utilization and integration of complementary and alternative medicine can be beneficial to reduce health care costs [15]. Accordingly, the aim of achieving lifelong health may involve educating the public through marketing and patient communications regarding the available options the chiropractic profession has to offer [6]. A change from the current health care paradigm to one that emphasizes prevention, lifelong wellness, and the empowerment of patients is well within the scope of the chiropractic profession.

Differentiation is an effective marketing strategy to ensure the chiropractic profession can carve out a niche within future health care markets. Within the context of CAM, the chiropractic profession and the individual practitioner must see the value of distinguishing the services they perform. Trust drives service differentiation, which drives commitment, which drives satisfaction and word-of-mouth marketing [16]. Market orientation involves the delivery of superior customer value, based on knowledge derived from customer and competitor analyses [17]. Knowledge of the health needs and desires of the individual, as well as knowledge of the unique health requirements of the community, has the potential to be a powerful differentiation tool. Moreover, the nature of the chiropractic visit involves direct patient contact and communications, encouraging a focus on high levels of service as a powerful differentiation tool.

The growth of CAM utilization rates has resulted in the chiropractic profession, experiencing usage rates in the population for 2008 measured at 5.2% [1]. Chiropractic is recognized as a CAM profession with core expertise of musculoskeletal (MSK) and spine conditions [18]. Chiropractic usage rates were 5.9% for persons 18–64 years old, 5.4% for patients over 65 with Medicare, and 3.1% for persons 18–64 with only public insurance [1]. Eighty-two percent of practicing chiropractors recognize the need for business education, with 16% indicating that they possess these skills [3]. The general business problem facing the profession is that current chiropractic training may not be preparing chiropractors to compete in the 21st century health care market [3]. Moreover, some chiropractors are experiencing a difficult time starting and successfully maintaining a practice [19].

Liu and Van Ginkel [20] posited that the public might not utilize chiropractic services due to the perception of ineffectiveness of treatments and services provided. Their study findings assert that the profession has not penetrated the public health care market with notable success. Underutilization of services can be the result if the public is not aware of the value added services the profession has to offer. Accordingly, business and marketing training can be effective to counteract misperceptions regarding the profession by usefully employing educational marketing. Such training develops valuable skills for building and enhancing patient loyalty towards the profession and the individual practitioner.

Penetrating into larger markets for health care services is similar to other business marketing in the 21st century, requiring the ability to appeal to a culturally diverse customer base. In the US, service quality has a stronger effect on customer satisfaction in the fast food industry when compared to China [21]. Smith and Reynolds [22] conducted a study to examine the differences between cognitive and affective measures for service quality, the ability to predict behavioral intentions, and the impact of service features on these measures. Their findings explained that customers perceived service quality and service satisfaction varied across different cultural groups.

Davis, Sirovich, and Weeks [23] stated that in 2006, 12.6 million adults in the US experienced 109 million chiropractic visits. This represents a 68% increase in chiropractic patient visits, and a 55% increase in total expenditures since 1997. The results suggests that individuals are seeking a relationship between the chiropractor and the patient that includes a broad perspective of health. Such an approach is in agreement with patient-centered care and sustainable approaches to health care delivery maintained by Bezold [24]. Weigel et al. [5] concurred with this finding in a longitudinal study that demonstrated an increased level of usage amongst older adults seeking preventative health care. Preventative and minimally invasive approaches to care are congruent with the worldview of chiropractors to relieve pain, and restore MSK function, while supporting the self-healing powers of the body [25]. Such preventative approaches can further contribute to sustainable health care within an environment of rapid change, creative innovation, and business opportunity.

## Methods

In this quantitative correlation study, a validated survey instrument (Health Care Training and Education Needs Survey) [3] was administered to chiropractors in the province of Ontario Canada via Survey Monkey. The eight items identify common business topics: organizational behavior/human resources, strategic management, finance, marketing, law and ethics, accounting, managerial decision-making, operations and systems management. Hypotheses statements were created for the eight items to test the relationship and significant difference between the perceived level of knowledge required and the perceived current level of knowledge for each of the eight business items: (a) organizational behavior and human resources (b) strategic management, (c) finance, (d) marketing, (e) law and ethics, (f) accounting, (g) managerial decision making, and (h) operations and systems management.

Permission to use Health Care Training and Education Needs Survey was obtained from the original author. The survey was used without modification as it had previously been administered to practicing chiropractors in the USA [3]. A panel of experts developed and validated the two questions and eight-item survey, and the instrument was previously used in two published peer-reviewed journal articles [3, 26]. The questions identify the knowledge required within the current health care market and the current level of knowledge possessed. Ethics approval was obtained through the IRB of Walden University.

Engagement of participants occurred with a briefly worded introduction to the survey to ensure cooperation and sincere participation to complete the entire questionnaire [27]. Participants accessed a 1–5 Likert-scale (5 – very high, 4 – high, 3 – neither high or low, 2 – low, 1 – very low) survey through an online link (Survey Monkey) with the option to respond over a 14-day period. Eight research questions were developed. Statistical analysis was performed to identify any significant correlations between the perceived level of knowledge required and the perceived level of existing knowledge for: a) organizational behavior and human resources, b) strategic management, c) finance, d) marketing, e) law and ethics, f) accounting, g) managerial decision making, and h) operations and systems management.

Spearman’s Rank Correlation (*r*_*s*_) was used to establish correlations between the eight items from survey Parts I and II. Spearman’s Rank Correlation is effective to determine the strength of association between two variables when using ordinal data [28]. Wilcoxon Rank Testing was selected to determine the statistically significant differences between variables when ordinal data is involved [28].

In Part I, the participants responded to the first question by rating the eight business items considering the current health care market and the level of knowledge required for each. In Part II, the participants respond to the second question by rating the eight items indicating their current level of knowledge for each area or subject. The eight items from both parts of the survey instrument matched the hypotheses developed for each item. Item matching permitted the process of hypotheses testing for each of the eight items [27].

Data collection began on November 27, 2013 and continued until December 18, 2013. The survey was closed after responses over the last four days of the three week period produced a rate of one or less responses per day. Initially, the data collection period was to occur over a two week period. However, during the first two weeks of the collection process, a larger than usual rate of non-completion was noticed. Accordingly, the data collection process was extended for another seven days. The initial survey was distributed to 2,391 eligible participants. The survey was distributed again one week following the initial survey as a reminder to those that had not completed. A third and final distribution of the survey occurred two weeks following the initial release date.

Data was also analyzed using the Bonferroni method to control for the Type I error rate due to running multiple bivariant tests [28]. The statistical analysis was performed using SPSS version 21. The frequency of participant responses (percentage) for perceived knowledge required and perceived current knowledge was performed. Henson et al. [3] used a similar technique of combining the *high* (4) and *very high* responses (5) for knowledge required and current knowledge followed by calculations to determine the percentage differences between the eight paired items.

## Results

From the approximately 4,000 chiropractors within the province of Ontario, 2,443 had their e-mails listed within the College of Chiropractors of Ontario (CCO) 2012–2013 directory. From this number of initially distributed surveys, 45 opted out, and seven e-mail addresses bounced. From the 361 respondents, 87 individuals did not complete the entire survey, including 15 individuals that elected to not continue beyond the consent page. This resulted in 274 completed and usable surveys (response rate of 11%) for data analysis purposes. The consent page clearly indicated that participation was voluntary and that discontinuing at any time was acceptable.

There was a statistically significant correlation between the level of knowledge required and the existing level of knowledge for organizational behavior and human resources, strategic management, marketing, law and ethics, managerial decision-making, and operations and systems management. There was no statistically significant correlation between the level of knowledge required and current level of knowledged possessed for finance and accounting. Effect sizes for finance and accounting were small: below 0.10. A moderate effect size was noted for law and ethics at 0.334. Organizational behavior, strategic management, marketing, managerial decision-making, operations and systems management had effect sizes ranging from low (0.10) to moderate (0.30) levels. The research findings confirmed that there are statistically significant relationships for six out of eight business items tested.

Table 1 contains the mean and standard deviation for each of the eight variables for knowledge required and current knowledge. The mean value ranges for knowledge required was 3.10 to 3.61; a difference of 0.51. The mean values range for current level of knowledge was 2.92 to 3.38; a difference of 0.46. The mean value for perceived knowledge required was slightly larger than perceived current knowledge supporting other data trends within this study.

**Table 1:** Descriptive statistics: Knowledge required and current knowledge (*N* = 274)

Eight business items	Knowledge required		Current knowledge	
	*M*	*SD*	*M*	*SD*
Strategic management	3.61	1.172	3.32	0.956
Marketing	3.57	1.163	3.15	0.986
Accounting	3.51	1.193	3.32	1.017
Organizational behavior	3.51	1.267	3.38	0.973
Operations	3.16	1.119	3.00	0.963
Legal and ethical	3.59	1.045	3.25	1.011
Managerial decisions	3.10	1.070	2.92	0.982
Finance	3.53	1.152	3.37	0.905

Table 2 contains the percentage differences of all participants for knowledge required in relation to current knowledge. Strategic management and marketing had the largest difference between knowledge required and current knowledge with a value of 20.1%. Accounting and organizational behavior were the next two items with the greatest value differences of 15.7% and 15.3%, respectively. Operations and systems management was next with a value difference of 13.8%, followed by legal and ethical at 13.1%, managerial decision-making at 9.4%, and finance at 9.2%.

**Table 2:** Percentage differences of all participants for knowledge required in relation to current knowledge

Eight			
Business items	Knowledge required	Current knowledge	Difference
Strategic management	65.7	45.6	20.1
Marketing	59.5	39.4	20.1
Accounting	61.3	45.6	15.7
Organizational behavior	63.5	48.2	15.3
Operations	43.8	30.0	13.8
Legal and ethical	55.4	42.3	13.1
Managerial decisions	37.2	27.8	9.4
Finance	58.1	48.9	9.2

Table 3 contains the correlation coefficients for the eight variables providing evidence for testing the relationship between the perceived level of knowledge required and perceived current level of knowledge. Table 3 also contains the *P* values for the Wilcoxon Signed Ranks Tests performed on each of the items. Six items demonstrated a statistically significant correlation between the perceived level of knowledge required and the perceived current level of knowledge: organizational behavior, strategic management, marketing, legal and ethical, and operations and systems management (*p* < 0.01). For finance and accounting, no statistically significant relationship was observed. For the Wilcoxon Signed Ranks Tests, three items demonstrated a significant difference (*p* < 0.01): strategic management; marketing and; legal and ethical. These three items demonstrated significantly higher scores for perceived knowledge required when compared to perceived current level of knowledge.

**Table 3:** Spearman’s correlations and Wilcoxon signed ranks tests: Knowledge required in relation to current knowledge

Eight	Spearman’s	Wilcoxon signed
Business Items	Correlation	Ranks Test (*P*-Values)
Strategic management	0.238*	<0.001
Marketing	0.222*	<0.001
Accounting	0.074	0.042
Organizational behavior	0.263*	0.073
Operations	0.260*	0.046
Legal and ethical	0.344*	<0.001
Managerial decisions	0.172*	0.029
Finance	0.048	0.055

*significant to *p* < 0.01

## Discussion

The study findings suggest that chiropractors from Ontario revealed a relationship between perceived knowledge required and current level of knowledge. This applies in particular to six of the eight items tested using Spearman’s correlation: organizational behavior, strategy, marketing, legal and ethical, managerial decisions, and operations and systems management. All significant correlations failed to reach a moderately positive level of 0.5 [28]. The items finance and accounting were not found to be statistically significant. The items strategic management, marketing, legal and ethical demonstrated Wilcoxon Signed Ranks Test significant differences suggesting a requirement to increase business knowledge for these topics.

Evans, Perle, and Ndetan [29] examined the websites of chiropractors advertising wellness. Their findings demonstrated that the majority of sites contained little useful health and wellness information and were instead used to deceitfully market the practice. Unethical marketing and business practices can destroy the cultural authority of a profession. Gleberzon, Perle, and LaMarche [19] pointed out that chiropractic curriculum should include business, practice management skills, and patient management skills with a focus on ethics. Gleberzon [30] recommended that chiropractic education reflect a standardized approach to sound business practices. Findings from the present study support recommendations of other researchers with positive albeit weak correlations for six of eight business items. The significant findings from the Wilcoxon analysis: strategic management; marketing and; legal and ethical, also support recommendations from other research findings.

A past study conducted by Henson et al. [3] measured the difference between the combined high and very high values for the perceived level of business knowledge required and current level of knowledge demonstrating percentage gaps for all eight items aligning with the results from the present study. The differences between the perceived knowledge required and current level of knowledge from the present study, demonstrate similarities with the Henson at al study. However, the Henson et al, study did not perform inferential statistical analysis on the perceived level of knowledge required and the perceived current level of knowledge for the eight items.

Chiropractors’ typically practice as solo practitioners, (sole proprietorship), associates, or within partnership arrangements. These arrangements typically involve income generation from patient visits and the sale of products such as supplements and supportive medical devices. The results of the current study suggest that there are relationships and gaps in business education and training for chiropractors. Mirtz et al. [31] obtained data from non-practicing chiropractors illustrating that meeting overhead expense was a crucial factor to business success. Income expectations from a chiropractic practice were much lower than expected, making chiropractic a difficult career to earn an adequate income. The combination of these two aforementioned items contributed to the decision for some individuals to discontinue practicing within the profession.

In the province of Ontario Canada, the number of practicing chiropractors doubled from 1990 to 2004 [32]. Similar trends in the supply of licensed chiropractors occurred in the state of California from 1970 to 1998. Foreman and Stahl [32] noted that during this period, the number of licensed chiropractors within the state of California increased by 170%. This represented a significant increase from the general population that expanded by 65% during the same period. Practitioners that could not meet their operating costs viewed the profession as unviable to make an adequate living and subsequently discontinued maintaining their license to practice [32]. Increased health care market competitive pressures, student loan debt, and lack of business knowledge resulted in an inability to generate adequate income levels.

Chiropractic educational requirements contain guidelines that include the delivery of content related to jurisprudence and business management. The accreditation body for chiropractic education in Canada (Canadian Federation of Chiropractic Regulatory and Educational Accrediting Board: CFCREAB) requires institutions to instruct students on ethical marketing, and understanding the need to follow sound business practices [30]. To increase acceptance and understanding to all students, such content can take the form of traditional lectures and online delivery formats. Frost, Derby, and Haan [33] demonstrated that computer-assisted online content for business and management principles was a successful method to augment lecture-based content across multiple learning styles. Gleberzon [30] posited that chiropractic institutions lack a comprehensive model curriculum for business and management content. Such a lack of consistency can undermine the ability of the profession to self-govern, be detrimental to professional cultural authority, and does not promote sustainable health care solutions. The results from the current study could provide the basis to reform the curriculum of chiropractic of chiropractic colleges.

### Limitations

This study had inherent limitations in that it only included participants from one Canadian province (Ontario) as well as self-selection bias on the part of individuals in choosing to reply to the survey. Accordingly, the results are only applicable to the chiropractic profession with the province of Ontario.

## Conclusion

The goal of conducting this study was to investigate the relationship and significant difference between business knowledge required and current level of business knowledge of chiropractors. Although relationships were established for six of the eight business items only three of the eight items were statistically significant for differences between them. The quanititative nature of the survey instrument may not be the most effective method to gather information from the participants related to the topics. It is recommended that future studies use a mixed methods or qualitative research design to obtain data of greater value.

Improving the level of business knowledge for chiropractors may prove valuable to promote sustainable health care encompassing prevention and lifelong wellness strategies positively contributing to the cultural authority of the profession. Moreover, such training could add value to the profession as an attractive health care career thereby strengthing enrolment and business sustainabilty of chiropractic educational institutions. Proficiency in business education may also be advantageous to influence individuals, society, and governments to adopt a health care paradigm that is efficient, effective, patient-centered, and empowers patients toward prevention type behaviors.

## Abbreviations

CAM: Complementary and alternative medicine
CCO: College of Chiropractors of Ontario
CFCREAB: Canadian Federation of Chiropractic Regulatory and Educational Accrediting Board
IRB: Institutional review board
MSK: Musculoskeletal
RQ: Research question
SMT: Spinal manipulative therapy
